# Mandibular intraosseous pseudocarcinomatous hyperplasia: a case report

**DOI:** 10.1186/s13256-016-1052-y

**Published:** 2016-09-29

**Authors:** Andreas Fuchs, Stefan Hartmann, Karen Ernestus, Grit Mutzbauer, Christian Linz, Roman C. Brands, Alexander C. Kübler, Urs D. A. Müller-Richter

**Affiliations:** 1 Department of Oral and Maxillofacial Plastic Surgery, University Hospital Würzburg, Pleicherwall 2, 97070 Würzburg, Germany; 2 Institute of Pathology and Comprehensive Cancer Center, University Würzburg, Josef-Schneider-Straße 2, 97080 Würzburg, Germany

**Keywords:** Case report, Pseudocarcinomatous hyperplasia, Intraosseous, Mandible

## Abstract

**Background:**

Mandibular pseudocarcinomatous hyperplasia is a rare and generally benign pathology. We report on one of these rare cases.

**Case presentation:**

The case history of a 73-year-old white man stated that he had a carcinoma of the oropharynx, which was primarily treated with radiotherapy and chemotherapy 4 years prior. As a result of radiotherapy he developed an osteoradionecrosis of his mandible and a consecutive pathological fracture of his left mandibular angle. Subsequent osteosynthesis was performed with a reconstruction plate. When we first saw him, his reconstruction plate was partially exposed with intraoral and extraoral fistulation. The resected bone of his defect-bordering jaw showed the typical pathohistological findings of an intraosseous mandibular pseudocarcinomatous hyperplasia. After a first reconstruction attempt with an iliac crest graft failed, definitive reconstruction of his mandible with a microvascular anastomosed fibula graft was achieved.

**Conclusions:**

Intraosseous pseudocarcinomatous hyperplasia of the mandible is a rare differential diagnosis in maxillofacial surgery. Besides other benign epithelial neoplasms, such as calcifying epithelial odontogenic tumor, squamous odontogenic tumor, or different forms of ameloblastoma, the far more frequent invasive squamous cell carcinoma needs to be excluded. A misinterpretation of pseudocarcinomatous hyperplasia as squamous cell carcinoma must be avoided because it can lead to a massive overtreatment.

## Background

Mandibular pseudocarcinomatous hyperplasia (PH) is a rare and generally benign disease, which is characterized by the proliferation of squamous epithelium without histological or cytological signs of malignancy. Clinical resemblance to oral squamous cell carcinoma (SCC) and other squamous neoplasms makes PH an important differential diagnosis in the field of maxillofacial surgery [1]. When PH is misdiagnosed as one of these pathologies it can have far-reaching implications for therapy and outcome. This particularly concerns a confusion of PH with SCC. Because of the high risk of local recurrence, lymphatic spread, or systematic metastasis, SCC requires an extended multimodal therapy whose foundation up to the present day is surgery [2]. Subject to the local extend and the involvement of adjacent anatomical structures, tumor resection in the head and neck area often results in large defects of soft tissue and hard tissue. In the case of intraosseous mandibular pathologies this can lead to the loss of large parts of the, or even the whole, mandible. At present, there are numerous techniques available to reconstruct these defects which can be broadly divided into local, regional, and distant tissue transplants often combined with alloplastic components for their structural support [3]. For the reconstruction of larger parts of the mandible there are basically three different types of microvascular osteocutaneous transplants available: the scapula flap, the iliac flap, and the fibula flap. The preparation and anastomization of all these bone grafts is complicated and time intensive [4]. Beyond that – depending on the chosen reconstruction technique – tissue replacement is almost always accompanied by different complications. These include: donor site morbidity at the place of transplant removal; impairment of respiration, mastication, swallowing, speech, and aesthetics; or implant failure [5, 6]. As the presence of PH requires a far less extended therapeutic approach with only local resection it should always be taken into consideration when squamous epithelial proliferation within the mandible is observed. Here we report a case of intraosseous PH as a complication of chronic recurrent osteomyelitis.

## Case presentation

A 73-year-old white man presented in 2014 with a chronic fistulation of his left mandible. He had a history of primary hypertension, type 2 diabetes mellitus, and hypercholesteremia. Furthermore, in 2010 the diagnosis of a poorly differentiated SCC of his oropharynx was made: tumor stage cT4 cN2 cM0 (*TNM*, 7th edition, 2010). Clinical findings displayed an exophytic tumor portion, which derived from the right lateral wall of his oropharynx, grew into his right epiglottic vallecula, infiltrated the lingual epiglottis and exceeded the midline in the region of his tongue base. Due to the size of the tumor and at his request a tumor resection was not made. Hence, primary therapy was performed as combined radiochemotherapy. External beam radiation was performed with a fractionation of five times 1.8 Gy per week up to an overall dose of 72 Gy. This was supplemented by a simultaneous chemotherapy with cisplatin and 5-fluorouracil in the first and sixth week of radiotherapy. During the oncological follow up there were no signs of residual tumor or lymph node metastases.

**Fig. 1:**
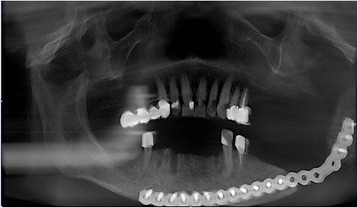
Preoperative cone beam computed tomography scan in May 2014. A reconstruction plate is used for stable bridging of the bone defect of the left mandibular angle

Due to an increasingly painful restricted mouth opening in 2013 he sought medical help again. At that time an intraoral fistula of the mucosa in his left retromolar region and a pathological fracture of his left mandibular angle on the basis of an osteoradionecrosis were detected. These conditions required a complete resection of his mandible in the retromolar region and the temporary bridging of the bony defect with a reconstruction plate (Fig. 1). Despite intensive wound care, wound healing only took place very slowly and remained insufficient with distinctive wound dehiscence.

**Fig. 2:**
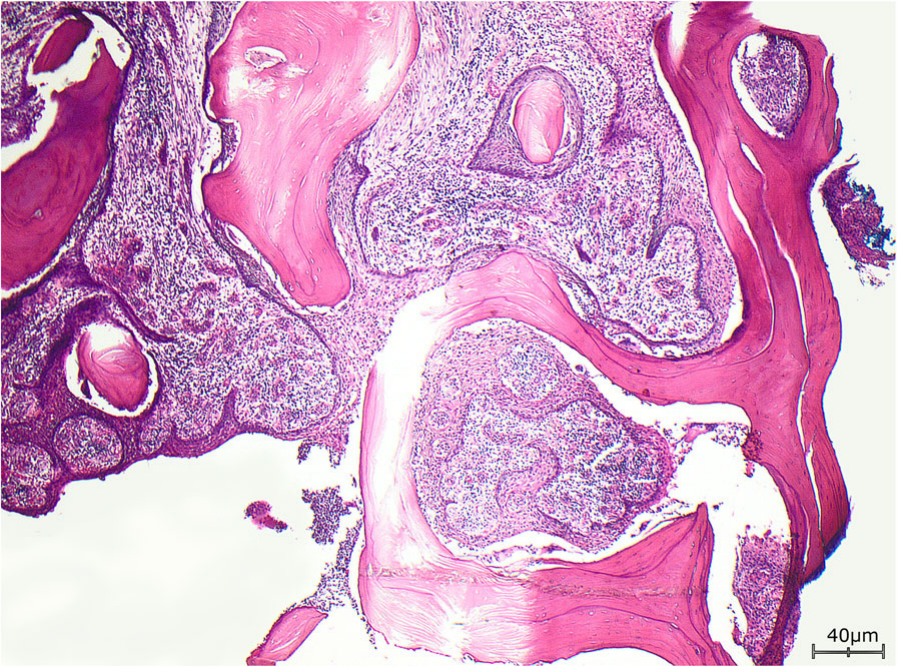
Intraosseous pseudocarcinomatous hyperplasia in response to chronic recurrent osteomyelitis. Hematoxylin and eosin stain, magnification ×40

**Fig. 3:**
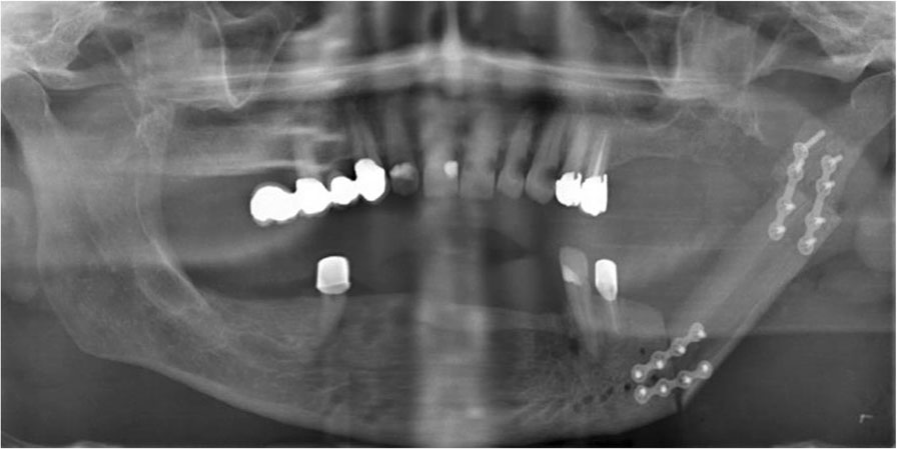
Postoperative panoramic scanning dental X-ray shows the fibula graft bridging the large bony defect of the left mandibular angle

After this he firstly attended our hospital in 2014 with exposed osteosynthesis material and persisting suppurating fistulation intraorally and extraorally with the desire for a definitive reconstruction of his mandible. A first reconstruction attempt with a microvascular anastomosed graft from his right iliac crest failed due to an insufficient venous anastomosis. The resected bone of his defect-bordering jaw showed the typical pathohistological findings of an intraosseous PH (Fig. 2). Five days later, after explantation of the iliac crest graft, the bony defect was successfully reconstructed with a microvascular anastomosed fibula graft along with an indicator flap from his right lower thigh (Fig. 3). The wound healing process was again extremely prolonged. Once more in spite of targeted antibiotic therapy with ciprofloxacin and extensive local wound care some long wound dehiscences developed, which healed only very slowly. After covering the skin defect of his right lower thigh with full thickness skin from his right lower abdomen and closure of the tracheostoma, he was discharged after 6 weeks in a good general condition. Seven months later, the occurrence of a pseudarthrosis at the anterior osteosynthesis required another surgical intervention including debridement of the bordering bone and re-osteosynthesis. By means of close monitoring and continuous local wound care all dehiscences were closed. His osteosynthesis material was finally removed after the bony continuity of his mandible was clearly documented intraoperatively. He is well except for a moderate painless restricted mouth opening and he has largely kept symptom-free.

### Pathologic findings

**Fig. 4:**
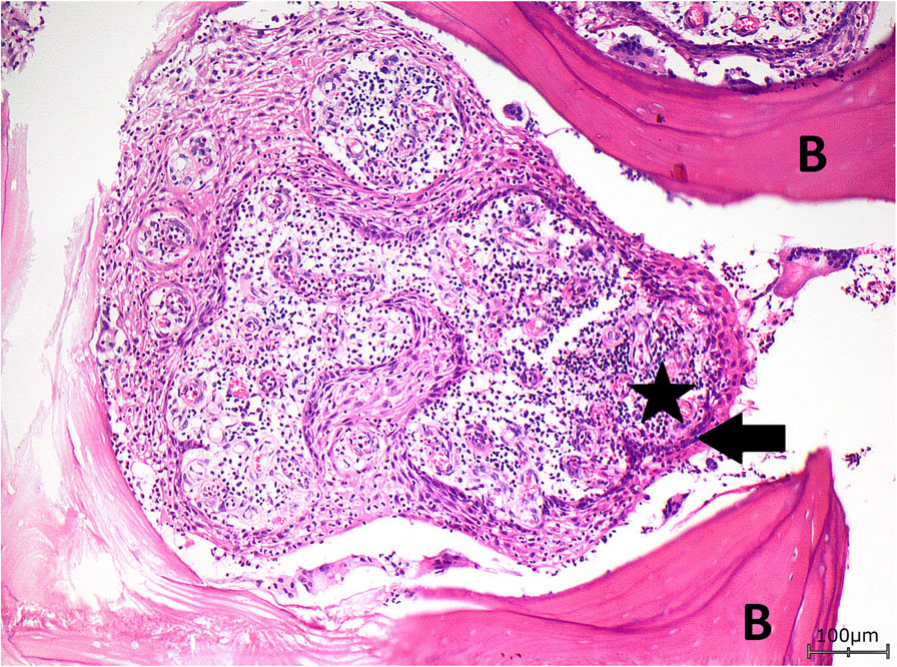
Pseudocarcinomatous hyperplasia. Bland-appearing squamous epithelial proliferates with no signs of malignancy (*arrow*) covering partially necrotic bone trabeculae (*B*) surrounded by a significant inflammation (*asterisk*; hematoxylin and eosin stain, magnification ×100)

**Fig. 5:**
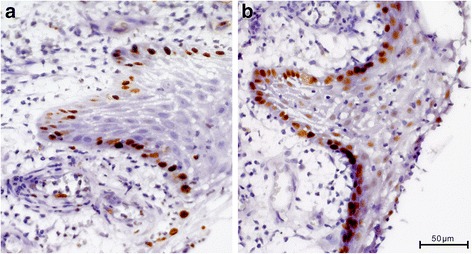
Immunohistochemical stainings of pseudocarcinomatous hyperplasia. **a** Ki-67 (magnification ×400), **b** p63 (magnification ×400)

**Fig. 6:**
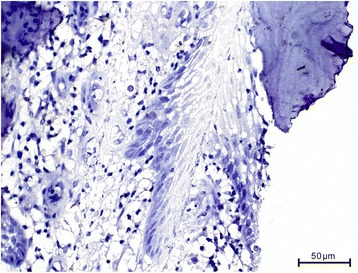
Immunohistochemical stainings of pseudocarcinomatous hyperplasia. Cytokeratin 7 (magnification ×400)

**Fig. 7:**
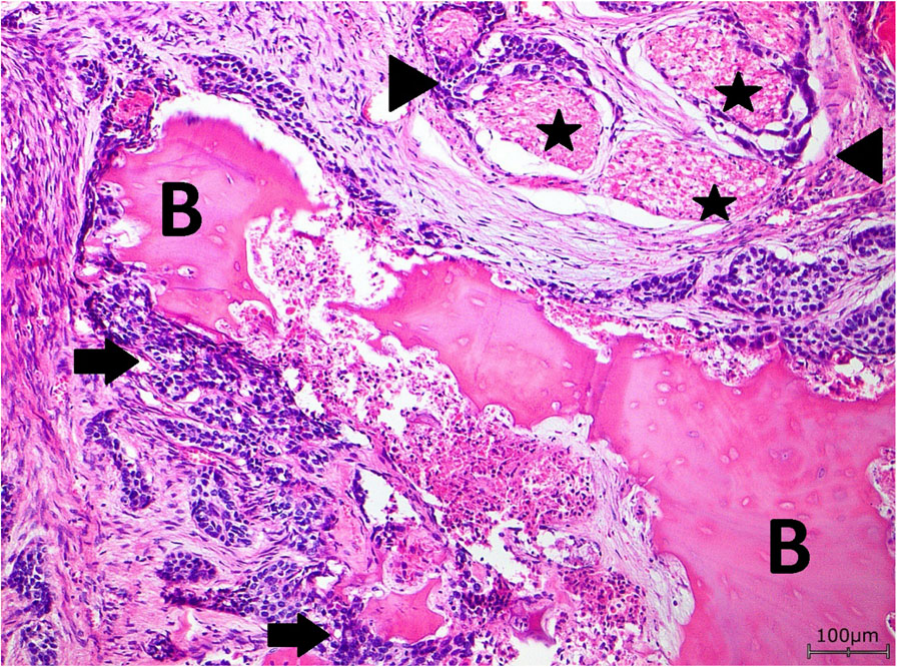
Bone invasion (*B*) and additional perineural invasion (*arrow heads*) by squamous cell carcinoma (*arrows*). Hematoxylin and eosin stain, magnification ×100

His bone tissue showed a chronic recurrent osteomyelitis. The trabecular bone was multifocally necrotic and surrounded by a mixed inflammatory infiltrate consisting of lymphocytes, plasma cells, and histiocytes. In addition, there were plenty of neutrophil granulocytes, focal formation of abscesses, and some bacterial colonies with features of *Actinomyces*. Furthermore, signs of bone reabsorption as well as bone remodeling and fibrosis of the medullary spaces were seen. The trabecular bone was partly covered by ribbon-like proliferations of regularly stratified squamous epithelium (Fig. 4) without nuclear atypia, mitosis, and dyskeratosis. Immunohistochemical stainings were inconspicuous. There was no overexpression of p53, and the proliferation rate, determined with the antibody against Ki67, showed a physiological staining pattern in the basal cell layers of the squamous epithelium (Fig. 5a). Moreover, the squamous epithelium expressed p63 (Fig. 5b), while cytokeratin 7 remained negative as expected (Fig. 6). Histological characteristics for the previously diagnosed SCC such as nuclear pleomorphism, increased mitosis, perineural invasion (Fig. 7), and an infiltrating growth pattern could not be detected.

In summary, this case is a rare example of an intraosseous PH in response to a chronic recurrent osteomyelitis.

## Discussion

PH is a rare pathologic entity, which is also known as pseudoepitheliomatous hyperplasia, invasive acanthosis, verrucoid epidermal hyperplasia, and carcinomatoid hyperplasia [7]. PH is a generally benign condition. Histological examination of the affected tissue shows a mainly thin and bland-appearing squamous proliferation, which tends to anastomose. Different degrees of papillomatosis and hyperkeratosis as well as a marked infiltration of inflammatory cells are also observed in PH [8]. Mitotic activity of cells is rare and the mitoses are not atypical. Keratinocyte necrosis or vascular and perineural infiltration cannot be found, whereas intraepithelial inflammatory cells such as neutrophils and eosinophils are common findings in PH [7].

As already mentioned, mandibular PH is an uncommon diagnosis and only very few reported cases can be found in the literature. The patient’s age, when first diagnosed with mandibular PH, varies widely between children, young adults, and older persons [1, 8]. PH is reported to be associated with several conditions such as infections (that is, mycobacterial and deep fungal infections), neoplasia (that is, basal cell carcinoma, granular cell tumor, lymphoma, dermatofibroma, and melanoma), irradiation (that is, as a consequence of a treatment of the neoplasia mentioned above), chronic inflammation and irritation (that is, hypertrophic lupus erythematosus, lichen simplex/sclerosus, halogenodermas, pemphigus, and pemphigoid), and other different processes (that is, associated with tattoo pigments) [7].

In all recently reported cases [1, 9, 10], mandibular PH was a complication of fistulated chronic osteomyelitis. It occurs after tooth extractions, the removal of radicular cysts, and segmentary mandibular resection with bone grafting as treatment of invasive plexiform unicystic ameloblastoma (PUA), acanthomatous ameloblastoma (AA), and solid and multicystic ameloblastoma. In cases of osteonecrosis, signs of wound repair such as cell migration, proliferation of epithelial cells, and re-epithelization can be observed. This occurs mostly at the margin of the bone defect [1, 8].

A discrete clinical pathology for PH of the mandible cannot be found in the common literature. Symptoms of patients diagnosed with PH, such as chronic pain and swelling with variable periods of exacerbation and improvement, are more likely to be attributed to an underlying disease such as chronic osteomyelitis. Nevertheless, oral PH has a distinct clinical presentation as a dome-shaped gingival swelling with a smooth or warty surface [9, 10].

A diagnosis of PH is mainly based on the evaluation of routine stains. Histological findings show partially necrotic bone trabeculae surrounded by proliferations of multilayered squamous epithelium without signs of cellular atypia such as dyskeratosis, atypical mitosis, or nuclear pleomorphism. Furthermore, immunohistochemistry, direct immunofluorescence, and tissue culture are additional diagnostic tools which can be used in contentious cases [7]. Immunohistochemical stainings such as p53, matrix metalloproteinase-1 (MMP-1), and E-cadherin have been proven to be valuable when it comes to distinguishing PH from well-differentiated SCC. In contrast to PH cells, invasive SCC cells show a decreased staining with E-cadherin and an increased staining with both p53 and, even more significantly, with MMP-1 [11]. In addition, radiological findings can be useful for the differential diagnosis of PH and SCC. Although they play only a minor role in the primary diagnostics of PH, radiographic examinations, such as hybrid single-photon emission computed tomography/computed tomography (SPECT/CT), can detect the existence and local extent of malignant bone infiltration of the mandible with very high sensitivity and specificity as well as a good delineation of the local tumor–bone contact area [12].

Distinguishing PH from a well-differentiated SCC arising from a long-lasting osteomyelitis can be difficult and requires careful investigation. Besides the lack of histological or cytological atypia, nuclear pleomorphism, massively increased mitotic rate, and dyskeratosis as seen in SCC, PH can also be differentiated from SCC regarding the duration of the lesions and their respective histological pattern. In contrast to SCC, PH shows a rather short duration of the lesions and a growth pattern with a centrifugal type of involvement of medullary spaces [1, 11]. But not only SCC has to be taken into consideration for the differential diagnosis of PH, several other benign squamous pathologies such as calcifying epithelial odontogenic tumor (CEOT), squamous odontogenic tumor (SOT), PUA, and AA have to be excluded before diagnosing PH [1]. CEOT is also a rare benign neoplasm of the mandible in adults which can be located either intraosseous or extraosseous. It manifests itself mostly as a single gingival painless tumor resembling a hyperplastic mucosal lesion. Histological findings of CEOT show mainly two different cell types embedded in a fibrous stroma: large polyhedral cells, which originate from intermediate squamous cells, and small basal-like cells, which can proliferate to form patterns like nests, cords, or acini [13]. SOT in turn is an infiltrative growing neoplasm which mimics the clinical appearance of localized periodontal disease and probably emerges from the rests of Malassez. On histological examination, it is composed of various small islands of differently sized, bland-appearing squamous epithelium with a moderately flattened or cuboidal peripheral basal cell layer surrounded by a collagenous fibrous connective tissue stroma [14]. PUA is a variant of ameloblastoma. In contrast to classic solid or multicystic ameloblastoma, it shows less infiltrative growth and a lower recurrence rate. PUA is characterized as a cystic-appearing lesion with an intraluminal proliferation of ameloblastic epithelium in the form of a plexiform pattern [15]. The term AA refers to the occurrence of extensive squamous metaplasia in central portions of the epithelial islands of follicular ameloblastoma, which is often associated with a certain degree of keratin formation [16].

Differentiation between reparative cytological atypia of PH and a well-differentiated SCC can be difficult and lead to misdiagnosis in some cases [17, 18]. Therefore it has been suggested by some authors that whenever squamous cell epithelium appears within bone, the diagnosis of SCC should be made and an amputation of the limb should be performed [1].

## Conclusions

Although mandibular PH is a disease pattern which is only rarely reported in the common literature, its appearance in the maxillofacial area is probably due to the proximity of the oral squamous epithelium to the alveolar bone. That is why mandibular PH should be taken into consideration, especially when the clinical suspicion of oral SCC arises, to prevent unnecessary diagnostics and therapy, such as resection of larger parts of the mandible, extended lymphadenectomy, and unnecessary chemoradiotherapy or radiotherapy.
